# Biopanning of allergens from wasp sting patients

**DOI:** 10.1042/BSR20181113

**Published:** 2018-10-17

**Authors:** Lin Chai, Xianyi Yang, Mei Liu, Chunyan Liu, Limei Han, Hui Guo, Changsheng Li, Yuwen Sun, Xiaoyan Li, Min Xiao, Zhicheng Fang

**Affiliations:** 1 Emergency Department, Taihe Hospital, Shiyan 442000, China; 2 Department of Pediatrics, Shiyan Maternal and Child Health-Care Hospital, Shiyan 442000, China; 3 Department of Pediatrics, Taihe Hospital, Shiyan 442000, China

**Keywords:** allergens, peptide, peptide, wasp venom

## Abstract

Objective: Wasp venom is a potentially important natural drug, but it can cause hypersensitivity reactions. The purpose of the present study was to systematically study the epitopes of wasp venom. Methods: Using a random 12-peptide phage library, we performed antibody-binding epitope panning on ten serum samples from wasp sting victims at 3 h and 4 days after the sting. The panning epitopes were identified by high-throughput sequencing and matched with wasp venom proteins by BLAST. The panned antibody-binding epitopes were verified by ELISA. Results: A total of 35 specific potential wasp venom epitopes in 4 days were identified. Amongst them, twelve peptide epitopes were matched with nine wasp venom proteins, namely, vitellogenin precursor, hexamerin 70b precursor, venom carboxylesterase-6 precursor, MRJP5, major royal jelly protein 8 precursor, venom acid phosphatase Acph-1 precursor, phospholipase A2, venom serine protease 34 precursor, and major royal jelly protein 9 precursor. The changes in serum IgM antibodies induced by wasp venom were confirmed by ELISA based on the 12 peptide epitopes. Conclusion: The nine wasp venom proteins are potential allergens, which should be excluded or modified in the potential biomedical applications of wasp venom.

## Introduction

There are more than 6000 kinds of wasps (Hymenoptera, *Vespoidea*) in the world and more than 200 kinds of wasps in China [1,2]. This diverse distribution is associated with a wide variation in wasp venom composition amongst the different genera [3]. Anaphylaxis caused by wasp venom can result in personal injury or death, and the incidence of morbidity is increasing, especially in industrialized countries [4]. The Rochester Epidemiology Project, in the United States, reported that the incidence of hypersensitivity reactions to wasp stings has increased from 21 in 100000 in the 1980s to the current 50 in 100000 [1]. The common Hymenoptera that can cause allergic reactions are mosquitoes, bees, and wasps, amongst others. Wasp toxins—which have important scientific value in the fields of pharmacology and immunology—are difficult to obtain.

The composition of wasp venom was first elucidated in the 1950s when scientists discovered that it was mainly composed of amines, peptides, enzymes, and other proteins with unknown function [5,6]. Venom is stored in the stinger glands, in the ventral end of the wasp, and is mainly composed of serotonin, histamine, bradykinin, hyaluronidase, and a large number of peptides and proteins [7–9]. Resulting from the development of modern molecular techniques, people now have more detailed knowledge about the components of wasp venom. Venom protein electrophoresis has revealed high protein concentrations at 23, 34, and 43 kDa, which have been identified as three major protein components of wasp venom: antigen-5 protein, phospholipase A1, and hyaluronidase, respectively [10–13]. In recent years, proteomic approaches have aided the delineation of wasp venom composition [7,14,15]. Danneels et al. [14] identified 53 proteins in wasp venom using Fourier transform ion cyclotron resonance MS (FT-ICR MS/MS). Matysiak et al. [15] identified 16 polypeptides using MALDI-TOF and nanoelectrospray quadrupole TOF (nanoESI-QqTOF) MS. By combing the LC-MALDI-TOF/TOF-MS and LC-ESI-QTOF-MS, Matysiak et al. [7] detected a total of 269 proteins, amongst them, 49 allergens involved in the mechanism of envenomation.

Wasp venom has potential clinical applications. It has important anti-inflammatory, bactericidal, antiviral, and antitumor effects [16–19]. It can also be used clinically for the treatment of rheumatoid arthritis and neurodegenerative diseases, such as multiple sclerosis, Alzheimer’s disease and Parkinson’s disease [20,21]. Despite this growing body of knowledge, specific wasp venom targets and mechanisms of allergic reaction remain unclear.

The pathological phenomena resulting from wasp stings and the clinical therapeutic effects suggest the involvement of a series of immune response inductions in the mechanism of allergic reactions [22,23]. It is speculated that a series of antibodies induced by a wasp sting has a therapeutic effect. However, there are no published investigations of antibodies specifically induced by wasp venom, nor their antigenic epitopes. In the present study, a random 12-peptide phage display technique was used to systematically study the epitopes bound with antibodies induced by wasp venom. This will help us to clarify the mechanism of hypersensitivity after stings and improve our ability to use wasp venom to treat immune diseases.

## Materials and methods

### Patients and serum specimens

Most patients presenting to our center after a wasp sting arrive 3 h after the sting. However, IgM is not produced until 3 days after a sting and is maximally expressed after 4 days. Therefore, serum was collected from ten patients (seven males and three females, with a mean age of 35.73 ± 18.26 years) with wasp sting injuries admitted to Taihe Hospital, Shiyan City, China between July 2015 and October 2017, at 3 h and 4 days after a wasp sting. We also collected serum from ten age- and sex-matched healthy controls (without pollen allergy or autoimmune diseases and who had not experienced a wasp sting within 5 years). The present study was approved by the ethics committee of Taihe Hospital, and all patients provided written informed consent before participating. The research was carried out in accordance with the Declaration of Helsinki.

### Randomized 12-peptide phage display technology for screening of wasp venom epitopes

We used a Ph.D.™-12 Phage Display Peptide Library Kit, catalog #E8110S, NEB. The screening procedure was as follows: sera collected at 3 h or 4 days were mixed. To each 100 μl aliquot of serum, we added 0.2 ml of protein A/G agarose solution (protein A:protein G, 1:1 by volume; protein A agarose, catalog #20334B; protein G agarose, catalog #20399B, Pierce, U.S.A.), mixed for 1 h at 37°C, and then washed ten-times with protein-buffered saline (PBS) with 1% Tween-20 (PBST), and ten-times with PBS to remove the unbound proteins. Then 1 ml of PBS and 10 μl of random 12-peptide phage (according to the manufacturer’s instructions, the phage complexity was approximately 10^9^ with a titer of approximately 10^12^) were added to the protein A/G-IgG/M/A. After 1 h at 37°C, ten washes with PBST, and ten washes with PBS, the bound phage was eluted and the total phage DNA was extracted and directly used in one-generation sequencing. The inserted random 12-peptide sequences were translated using PerlBio software. The same peptide sequences were combined, and the copy numbers were summed. The copy numbers of high abundance peptides (copy number > 50) accounted for 90% of the copy numbers of all peptides, so the low abundance peptides (copy number < 50) were removed. Then, the sequences between the two groups were compared, and specific sequences were achieved in the sera taken at 4 h.

Panning epitopes were identified by high-throughput sequencing and matched with bee and wasp venom proteins by BLAST with a database downloaded from the NCBI non-redundant protein sequence (nr) database. The species was selected as: Organism: *Apis mellifera* (taxid:7460). Matching conditions: 1, perfect match; 2, only one gap; 3, only one mismatch.

### ELISA

Peptides were synthesized by China Peptides Corporation, China. The synthesized peptides were purified and analyzed using HPLC. All synthetic peptides had a purity of at least 95%. Scramble peptide was used as a negative control and metlin polypeptide as a positive control. The synthesized peptides were dissolved in PBS, and the concentration of each peptide was 0.1 mg/ml. We added 100 μl of peptide solution to each well of 96-well ELISA plate (Cat #: 3590, Corning, U.S.A.), incubated at 37°C for 2 h and patted dry. The plates were blocked in 200 μl of 1% BSA for 2 h at room temperature and then patted dry. Each serum sample was diluted to five-fold with 1% BSA, then 100 μl was placed into ELISA wells, incubated at 37°C for 1 h, washed with 0.5% PBST ten times and PBS ten times, and incubated with 100 μl of mouse-anti-human IgM-HRP (1:2000 in 1% BSA) at 37°C for 1 h. After ten washes each with 0.5% PBST and PBS, 100 μl of tetramethylbenzidine was added for 15 min for chromogenic reaction. The reaction was terminated by adding 15 μl of 3% H_2_SO_4_ solution. Finally, a microplate reader (Biotech, U.S.A.) was used to read at 563 nm. Using a random peptide as a reference, an absorbance greater than twice that of the random peptide was considered positive.

### Statistical analysis

The statistical analyses of ELISA data were performed using SPSS 20.0 (IBM Corp., Armonk, NY, U.S.A.). *P*-values less than 0.05 were considered statistically significant.

## Results

### Screening of wasp venom antigen epitopes

After excluding the low-abundance peptides (copy number < 50), a total of 4356 peptides were achieved in the 3-h group, and 4408 peptides in 4-day group. We compared the peptides between the two groups, and 35 specific peptides were achieved in the 4-day group (Table 1).

**Table 1:** Thisrty-five specific peptides in serum of patients with wasp stung after 4 days

ID	Sequence	Read number
**Peptide 1**	**QVDTQGENAVKV**	**189**
**Peptide 2**	**PTVYHPELYQKA**	**187**
Peptide 3	AVMRQQTDELRL	186
Peptide 4	AVHSNLFPGQPD	185
Peptide 5	DPSDVLTLPFPR	183
**Peptide 6**	**FQFASGNEANET**	**181**
Peptide 7	WEIANPYWDGSE	170
**Peptide 8**	**VTVRENSPRKLA**	**166**
Peptide 9	YPNLLLLASVDV	166
**Peptide 10**	**QGVSDIHSRNLT**	**159**
Peptide 11	APAQPAESIHAY	155
Peptide 12	RVTAPRPEFSTL	147
Peptide 13	LPRVPPPVHSTT	143
Peptide 14	ALSKTFEVAPLH	142
Peptide 15	AYPSYLTSDGYH	141
Peptide 16	IDTQYPSAMTLT	140
Peptide 17	DIHRHVVGARTL	136
Peptide 18	TTMRIAFHQLHT	134
**Peptide 19**	**RGELTNSGKARE**	**134**
Peptide 20	HGRFPLTSDVPT	123
Peptide 21	SMPSMLFDTGED	121
Peptide 22	ACAATPLNCGG	119
**Peptide 23**	**QIRDRIHDNELE**	**116**
Peptide 24	VETIPPLRYSDP	110
**Peptide 25**	**SENKNCNAGSLT**	**102**
Peptide 26	QPPHIHSALTLM	101
Peptide 27	VAGTLPAPSPSY	90
**Peptide 28**	**NLGNYNDKEAVN**	**84**
**Peptide 29**	**HDWSSKTETNAT**	**84**
Peptide 30	FMNTHDRADLSI	81
Peptide 31	LLKHIEVSLPLA	80
Peptide 32	QWYHRSDGGGSA	70
**Peptide 33**	**AINSTTGKRNVV**	**61**
Peptide 34	LACAVTGLICGG	59
**Peptide 35**	**RKAHQEKDSPRI**	**51**
Positive control (melittin peptide)	AAPEPEPAPEPEAEADAEADPEAGI	
Negative control (scramble peptide)	QNILIHFASPSH	

Bold indicates the peptides matched with wasp venom proteins.

### Matching of identified antigenic epitopes with wasp venom antigens

In order to clarify the origin of these epitopes, we used the BLAST method to match these screened epitopes with the wasp protein database. Nine wasp venom proteins (Table 2) were matched with 12 screened peptides (red, Table 1). The nine wasp venom proteins were vitellogenin precursor [24], hexamerin 70b precursor [24], venom carboxylesterase-6 precursor [24], MRJP5 [25], major royal jelly protein 8 precursor [26], venom acid phosphatase Acph-1 precursor [27], phospholipase A2 [28], venom serine protease 34 precursor [6,24], and major royal jelly protein 9 precursor [26].

**Table 2:** The wasp venom proteins matched with screened peptides

Score	Expect	Identities	Positives	Gaps	Alin	Targets (*Apis mellifera*)	ID
40.1 bits (87)	0.000002	12/12 (100%)	12/12 (100%)	0/12 (0%)	Query 1 QVDTQGENAVKV 12	Vitellogenin precursor	Q868N5
					Sbjct 141 QVDTQGENAVKV 152		
43.5 bits (95)	1E-07	12/12 (100%)	12/12 (100%)	0/12 (0%)	Query 1 PTVYHPELYQKA 12	Hexamerin 70b precursor	Q6J4Q1
					Sbjct 48 PTVYHPELYQKA 59		
40.9 bits (89)	9E-07	12/12 (100%)	12/12 (100%)	0/12 (0%)	Query 1 FQFASGNEANET 12	Venom carboxylesterase-6 precursor	B2D0J5
					Sbjct 136 FQFASGNEANET 147		
40.1 bits(87)	0.000002	12/12 (100%)	12/12 (100%)	0/12 (0%)	Query 1 VTVRENSPRKLA 12	MRJP5	O97432
					Sbjct 21 VTVRENSPRKLA 32		
38.0 bits (82)	0.00001	11/12 (92%)	11/12 (91%)	0/12 (0%)	Query 1 QGVSDIHSRNLT 12	Major royal jelly protein 8 precursor	Q6TGR0
					Sbjct 20 QGVTDIHSRNLT 31		
34.6 bits (74)	0.0002	11/12 (92%)	11/12 (91%)	0/12 (0%)	Query 1 RGELTNSGKARE 12	Venom acid phosphatase Acph-1 precursor	Q5BLY5
					Sbjct 53 RGELTNSGKMRE 64		
40.1 bits (87)	0.000002	12/12 (100%)	12/12 (100%)	0/12 (0%)	Query 1 SENKNCNAGSLT 12	Venom serine protease 34 precursor	Q8MQS8
					Sbjct 90 SENKNCNAGSLT 101		
36.3 bits (78)	0.00004	11/12 (92%)	11/12 (91%)	0/12 (0%)	Query 1 QIRDRIHDNELE 12	Phospholipase A2	P00630
					Sbjct 20 QIRDRIGDNELE 31		
38.0 bits (82)	0.00001	11/12 (92%)	11/12 (91%)	0/12 (0%)	Query 1 NLGNYNDKEAVN 12	Hexamerin 70b precursor	Q6J4Q1
					Sbjct 67 NLDNYNDKEAVN 78		
42.2 bits (92)	3E-07	12/12 (100%)	12/12 (100%)	0/12 (0%)	Query 1 HDWSSKTETNAT 12	Venom serine protease 34 precursor	Q8MQS8
					Sbjct 220 HDWSSKTETNAT 231		
41.8 bits (91)	5E-07	12/12 (100%)	12/12 (100%)	0/12 (0%)	Query 1 RKAHQEKDSPRI 12	Vitellogenin precursor	Q868N5
					Sbjct 619 RKAHQEKDSPRI 630		
39.2 bits (85)	0.000004	12/12 (100%)	12/12 (100%)	0/12 (0%)	Query 1 AINSTTGKRNVV 12	Major royal jelly protein 9 precursor	Q4ZJX1
					Sbjct 175 AINSTTGKRNVV 186		

### ELISA validation of antibody–antigenic epitopes reactions

**Figure 1:**
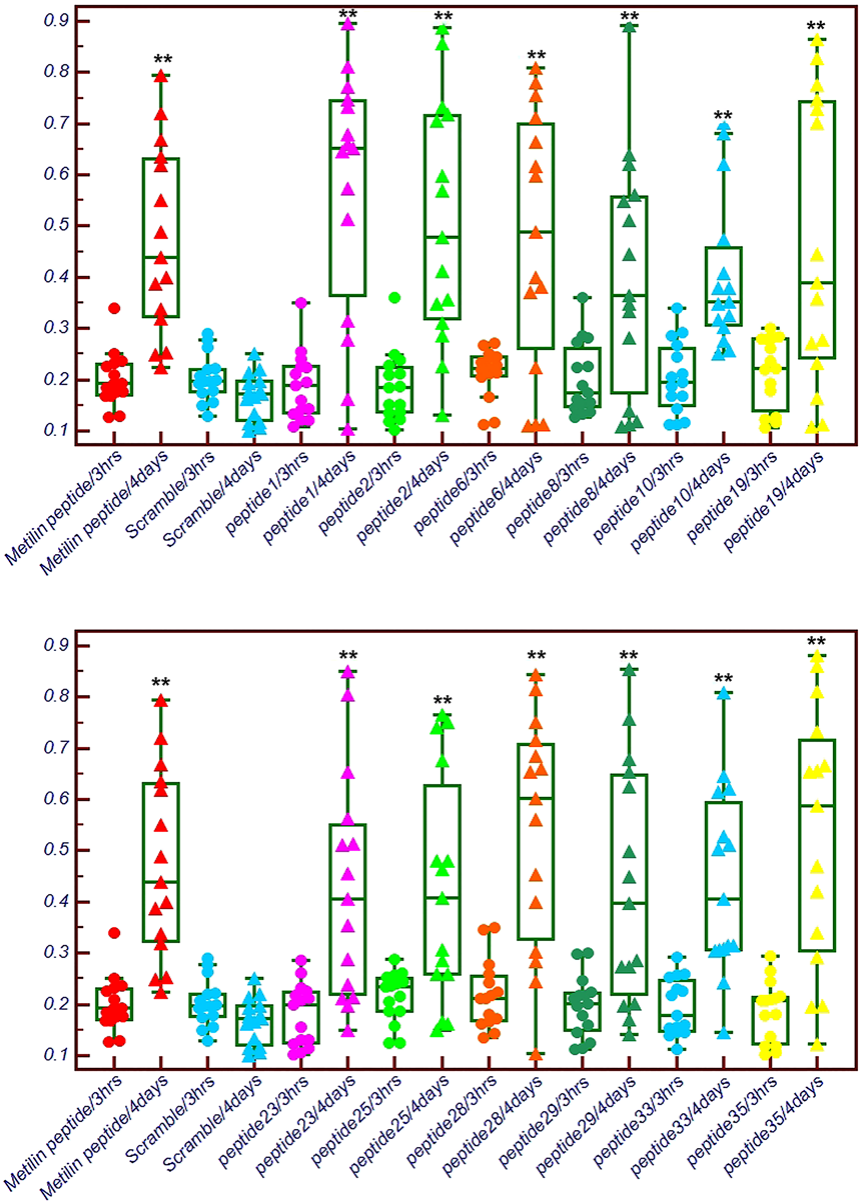
ELISA detection of the IgM in serum of patients stung by wasp after 3 h or 4 days with 12 antigenic epitopes **P<0.01.

In order to verify the 12 antigenic epitopes matched with wasp venom proteins, we synthesized the peptides and immobilized them on an ELISA plate to make an ELISA kit to detect IgM in the sera of patients stung by wasps 3 h or 4 days earlier (Figure 1). All 12 peptides (peptides 1, 2, 6, 8, 10, 19, 23, 25, 28, 29, 33, and 35) were expressed at significantly higher amounts after 4 days than after 3-h group. The positivity rates of all peptides to antibodies were between 60 and 85% (Table 3). The false positivity rates were all less than 7%, except for peptide 28 which had a 13% false positive rate.

**Table 3:** Positive rate and false positive rate of peptides in wasp patients’ serum

Peptides	Positive control	1	2	6	8	10	19	23	25	28	29	33	35
Positive rate	0.73	0.73	0.73	0.73	0.67	0.6	0.6	0.6	0.53	0.73	0.53	0.53	0.73
False positive rate	0.07	0.07	0.07	0	0.07	0.07	0	0	0	0.13	0	0	0

## Discussion

Wasp venom is a multicomponent naturally occurring product, mainly composed of peptides and proteins. In recent years, with the emergence of high-sensitivity proteomics detection technology, the polypeptide and protein components have been gradually revealed. There are a total of approximately 50 reported peptides and proteins [7,15,29]. Clinical symptoms and pathological studies carried out on wasp sting victims have demonstrated that wasp toxins induce a strong immune response; studies have also identified some of the important allergens [30–32]. In this study, we used a random 12-peptide phage library display technique and screened 35 potential epitopes or analog epitopes.

Using BLAST, we matched 12 peptides with nine wasp venom proteins. Another 23 peptides were not directly matched with wasp venom proteins, and these may have been analog epitopes. Using ELISA, we confirmed that the 12 peptides matched with wasp venom proteins reacted with IgM in the serum samples of the patients with wasp stings (after 4 days). However, not all patients produced antibodies against those epitopes. The positivity rate of each epitope to antibodies was between 60 and 85%. Currently, most people pay more attention to the IgG and IgG antibodies induced by wasp venom. For example, it was demonstrated that serum IgE and IgG concentrations increased significantly during wasp sting-induced immune responses after 3 h and that IgE was associated with allergies, perhaps being the main factor causing allergic reactions after wasp stings [30,33–35]. In the present study, to screen new epitopes, we focussed on 4 days after the wasp sting and detected the IgM indicators. The antibodies produced during the 4-day interval showed a lower specificity or lower affinity to those epitopes, which might avoid the reaction of other antigens in the body during the 2 weeks that produce the highest amount of IgG.

Concerning the biological functions of the wasp venom, melittin peptide is currently the most studied [16,18]. It was demonstrated that melittin plays roles in killing tumor cells [36], anti-inflammation [37,38], and improving the microcirculation [39]. All of these functions involve the injection of these proteins into the blood, so how to avoid the immune system’s rejection of these proteins is a matter that should be considered. Whether the wasp venom proteins identified in the present study (vitellogenin precursor, hexamerin 70b precursor, venom carboxylesterase-6 precursor, MRJP5, major royal jelly protein 8 precursor, venom acid phosphatase Acph-1 precursor, phospholipase A2, venom serine protease 34 precursor, and major royal jelly protein 9 precursor) induced the body to produce IgM antibodies correspondingly needs to be investigated in the future.

According to reports in the literature, allergic reactions to wasp venom are common after stings [40]. At present, wasp venom antigen is used as a primary antigen in clinical skin allergy tests [32,41]. However, the severities of sting reactions have shown a lack of association with skin testing findings, venom-specific IgE levels, and molecular diagnoses [42]. The discovered antigens in this study have a potential predictive value for clinical severity in wasp sensitization.

Due to the low incidence of wasp stings, it was impossible to collect more clinical samples for experimentation. This is an important limitation of the present study. Moreover, future research should detect the specific IgG and explore how many IgM-producing B cells transferred to express IgG. Additionally, in the present study, we focussed on the epitopes associated with wasp venom; the panning epitopes were identified by high-throughput sequencing and matched with wasp venom proteins by BLAST. We did not detect other pathogens. Important information might be found by matching the epitopes with other protein databases.

In summary, nine wasp venom proteins, namely, vitellogenin precursor, hexamerin 70b precursor, venom carboxylesterase-6 precursor, MRJP5, major royal jelly protein 8 precursor, venom acid phosphatase Acph-1 precursor, phospholipase A2, venom serine protease 34 precursor, and major royal jelly protein 9 precursor were identified. These all have potential uses in immunotherapy and predicting the clinical severity of hypersensitivity reactions to wasp stings.

## Abbreviations

PBS: protein-buffered saline
PBST: PBS with 1% Tween-20
